# Right Under Your Nose! A Search for a Dislodged Tooth Discovered in the Nasopharynx by Lateral Neck Radiograph

**DOI:** 10.7759/cureus.83201

**Published:** 2025-04-29

**Authors:** Christopher R Parrino, Ron E Samet

**Affiliations:** 1 Anesthesiology, University of Maryland School of Medicine, Baltimore, USA

**Keywords:** aspiration, foreign body, lateral x-ray, missing tooth, nasopharynx

## Abstract

During nasotracheal intubation for mandibular fixation surgery in an 85-year-old man, a loose tooth was inadvertently dislodged and remained undetected despite repeated laryngoscopic and bronchoscopic exploration. Antero-posterior chest, abdominal, and neck x-rays also failed to locate the tooth. A lateral neck radiograph was then obtained, which revealed the tooth in the nasopharynx. A nasopharyngeal airway was inserted, enabling the tooth to be successfully displaced caudally and extracted. This case highlights the value of lateral neck x-rays in identifying foreign bodies, especially in supine patients where objects may fall posteriorly and cephalad into the nasopharynx.

## Introduction

Foreign bodies in the airway pose significant challenges due to their potential to migrate into large airways, causing obstruction or other respiratory complications [1,2]. Rapid identification and controlled retrieval of such foreign bodies is crucial. Radiographs can be instrumental when direct visualization fails to locate a foreign body [1,2]. This case report details the search for a dislodged tooth that could not be found via laryngoscopy, bronchoscopy, or antero-posterior (AP) neck, chest, or abdominal x-rays. Ultimately, a lateral neck x-ray identified the tooth in the nasopharynx, facilitating its retrieval. Including lateral radiographic films during the initial search for a foreign object in the airway is advised. The patient provided written informed consent for publication by Health Insurance Portability and Accountability Act (HIPAA) guidelines. This report adheres to the CARE guidelines for case reports. 

## Case presentation

**Figure 1:**
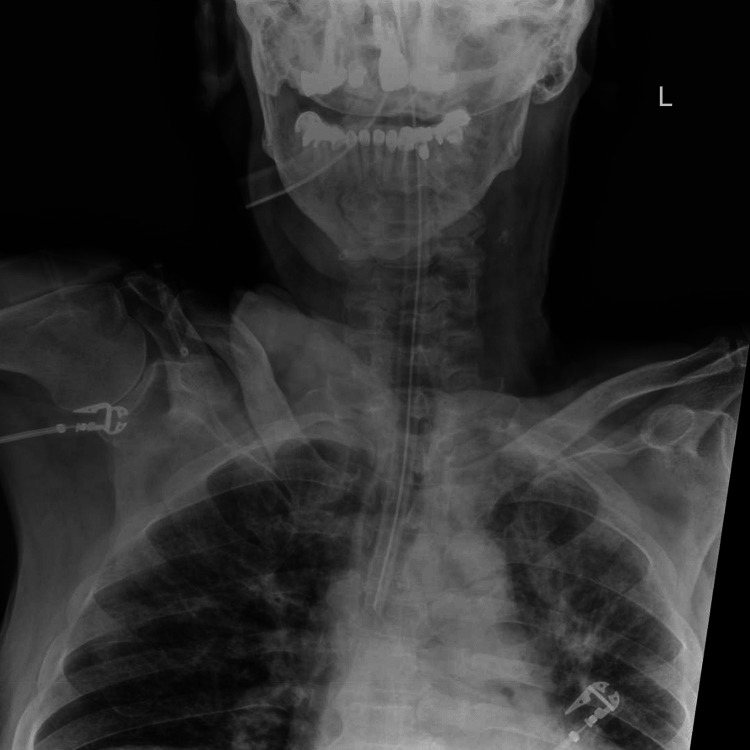
Antero-posterior (AP) radiograph of the neck and upper chest. This radiograph was obtained to localize a dislodged radiopaque tooth. No solitary tooth is identified in the mouth, neck, or upper chest.

This case involves an 85-year-old American Society of Anesthesiologists (ASA) III male who sustained bilateral subcondylar mandibular fractures and multiple loose teeth following a ground-level fall. He was scheduled for open reduction and internal fixation of the mandible. The preoperative anesthetic evaluation noted two loose upper frontal incisors; Mallampati score was unable to be assessed due to limited mouth opening in the setting of pain. Nasotracheal intubation was performed using video laryngoscopy and was complicated by the inadvertent dislodgement of a loose front tooth. Although the patient was successfully nasotracheally intubated through the left nare, extensive examination of the oropharynx and airway using direct laryngoscopy, video laryngoscopy, and bronchoscopy failed to locate the tooth. AP x-rays of the neck, chest, and abdomen also did not reveal the tooth's location (Figure 1). The surgery proceeded as planned, but the foreign body remained undetected within the oral cavity throughout the four-hour procedure. To prevent possible aspiration in the setting of the still-missing foreign body, the patient remained intubated postoperatively.

**Figure 2:**
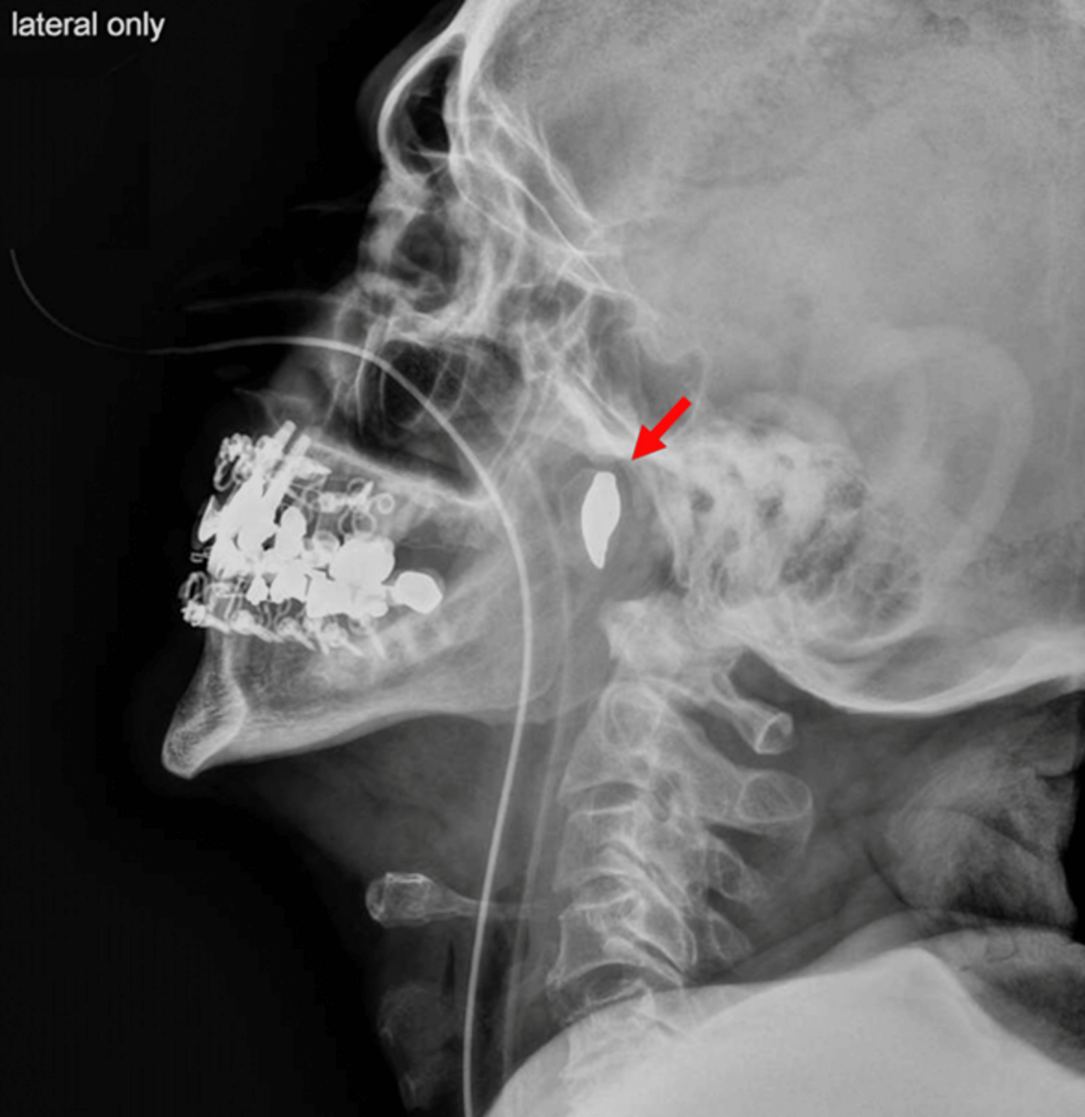
Lateral radiograph of the neck demonstrating a solitary radiopaque tooth in the nasopharynx

Following the uneventful surgery, a lateral neck radiograph was suggested as a final attempt to locate the tooth. This imaging revealed the tooth in the nasopharynx, positioned posterior and cephalad to the soft palate (Figure 2). A nasopharyngeal airway was inserted into the patient's right nares, advancing the tooth caudally into the oropharynx, where it was promptly visualized and removed.

Postoperatively, the patient was kept intubated due to multiple instances of airway instrumentation and was administered dexamethasone to manage potential airway edema. He was successfully extubated six hours after the procedure and discharged home on postoperative day 2 as planned. The incident was disclosed to the patient, who expressed understanding, given that he had multiple loose teeth resulting from the fall.

## Discussion

Dental injury during anesthesia care occurs rarely but is the leading cause of anesthesia-related malpractice claims [3,4]. The incidence of anesthetic-related dental injury ranges from 0.02% to 0.1%, depending on the definition [4-8]. The risk of dental injury is increased more than threefold in patients with pre-existing poor dentition [4]. Risk factors for dental injury include poor dentition, difficult laryngoscopy, difficult intubation, and use of video laryngoscopy [8]. Our patient already had loose teeth as a result of his fall, placing him at higher risk for tooth dislodgement. Timely retrieval of dislodged foreign bodies is crucial to prevent potential complications such as aspiration, bronchospasm, pneumonitis, airway obstruction, or esophageal injury [1,2]. Selecting the appropriate imaging technique for foreign body identification enables clinicians to plan the safest and most effective retrieval method.

While chest x-rays and bronchoscopy are valuable, they can sometimes fail to identify a dislodged tooth when it settles in the upper airway or is obscured by other anatomical structures. Chest x-rays may not provide sufficient detail of the upper cervical region, and AP neck x-rays may offer an insufficient view of the posterior pharynx due to obscuration by radiopaque teeth as seen in Figure 1.

In this patient, the dislodged tooth was located cephalad to the oropharynx, preventing detection via direct visualization or laryngoscopy. The anterior neck x-ray did not differentiate between the existing anterior teeth and the posterior solitary tooth. The lateral neck x-ray provided the necessary distinction between anterior and posterior structures, which is especially valuable when a patient is lying supine and foreign bodies may be displaced posteriorly and cephalad to the soft palate.

The initial search process in the OR, including radiographic imaging and fiberoptic bronchoscopy, totaled about 45 minutes and was unsuccessful. As a result, the patient's upper airway was instrumented multiple times, and the time under general anesthesia was extended. If a lateral radiograph had been included as part of the initial search, as it shall be at our institution going forward, the tooth would have been immediately localized, thereby saving this patient additional airway instrumentation attempts and time under a general anesthetic.

Case reports of unexpected nasopharyngeal foreign bodies and their detection are primarily limited to the pediatric population due to their proclivity to ingest foreign objects [9,10]. One case report detailed inadvertent dislodgement of a tooth during maxillofacial surgery in a six-year-old female that was similarly only able to be detected by lateral neck radiograph [11]. Another report describes a displaced incisor noted in the nasopharynx by cervical spine x-ray, but this tooth passed spontaneously into the gastrointestinal tract prior to induction of anesthesia [12]. One patient had a four-unit dental bridge that was displaced into the nasopharynx during intubation and required removal by the otorhinolaryngology service [13]. Lateral neck x-ray proved useful in the detection of a maxillary incisor displaced after multiple failed attempts at intubation in another patient [14]. Rarely, advanced techniques such as nasal endoscopy may be required to facilitate the removal of deeply wedged foreign bodies from the nasopharynx [15]. In a recent study, about 80% of anesthetists report having encountered peri-anesthetic dental trauma during their career [16], further underscoring the importance of detection techniques such as the one described in this report.

## Conclusions

Dislodged teeth are an unusual but important complication of laryngoscopy due to their potential to cause damage or obstruction if not retrieved. This case adds to the paucity of anesthesiology literature about alternative techniques, such as lateral neck x-ray, for identification of misplaced foreign bodies. Upon suspicion of the presence of a foreign body, visual inspection of the oropharynx should be undertaken, followed by video laryngoscopy and AP and lateral x-rays of the face, neck, chest, and abdomen. If the foreign body is still unable to be identified, fluoroscopy or computed tomography should be considered. Given the quick, feasible, and non-invasive nature of obtaining a radiograph, it is advisable to include lateral films in the initial exploratory modalities for locating foreign objects in the airway. Institutions with protocols for identification and retrieval of lost foreign bodies should consider the addition of lateral x-rays to their search protocol.
